# Molecular Evolution of HIV-1 CRF01_AE Env in Thai Patients

**DOI:** 10.1371/journal.pone.0027098

**Published:** 2011-11-02

**Authors:** Samatchaya Boonchawalit, Duangrat Jullaksorn, Jiraporn Uttiyoung, Amara Yowang, Nongkran Krathong, Sununta Chautrakul, Akifumi Yamashita, Kazuyoshi Ikuta, Amornsak Roobsoong, Sangkom Kanitvittaya, Pathom Sawanpanyalert, Masanori Kameoka

**Affiliations:** 1 Thailand-Japan Research Collaboration Center on Emerging and Re-emerging Infections (RCC-ERI), Nonthaburi, Thailand; 2 National Institute of Health, Department of Medical Sciences (DMSc), Ministry of Public Health (MOPH), Nonthaburi, Thailand; 3 Regional Medical Science Center Chiangrai, DMSc, MOPH, Chiangrai, Thailand; 4 Mae-Soay Hospital, Chiangrai, Thailand; 5 Graduate School of Life Science, Tohoku University, Miyagi, Japan; 6 Research Institute for Microbial Diseases, Osaka University, Osaka, Japan; McGill University AIDS Centre, Canada

## Abstract

**Background:**

The envelope glycoproteins (Env), gp120 and gp41, are the most variable proteins of human immunodeficiency virus type 1 (HIV-1), and are the major targets of humoral immune responses against HIV-1. A circulating recombinant form of HIV-1, CRF01_AE, is prevalent throughout Southeast Asia; however, only limited information regarding the immunological characteristics of CRF01_AE Env is currently available. In this study, we attempted to examine the evolutionary pattern of CRF01_AE Env under the selection pressure of host immune responses.

**Methodology/Principal Findings:**

Peripheral blood samples were collected periodically over 3 years from 15 HIV-1-infected individuals residing in northern Thailand, and amplified *env* genes from the samples were subjected to computational analysis. The V5 region of gp120 showed highest variability in several samples over 3 years, whereas the V1/V2 and/or V4 regions of gp120 also showed high variability in many samples. In addition, the N-terminal part of the C3 region of gp120 showed highest amino acid diversity among the conserved regions of gp120. Chronological changes in the numbers of amino acid residues in gp120 variable regions and potential N-linked glycosylation (PNLG) sites are involved in increasing the variability of Env gp120. Furthermore, the C3 region contained several amino acid residues potentially under positive selection, and APOBEC3 family protein-mediated G to A mutations were frequently detected in such residues.

**Conclusions/Significance:**

Several factors, including amino acid substitutions particularly in gp120 C3 and V5 regions as well as changes in the number of PNLG sites and in the length of gp120 variable regions, were revealed to be involved in the molecular evolution of CRF01_AE Env. In addition, a similar tendency was observed between CRF01_AE and subtype C Env with regard to the amino acid variation of gp120 V3 and C3 regions. These results may provide important information for understanding the immunological characteristics of CRF01_AE Env.

## Introduction

Human immunodeficiency virus type-1 (HIV-1) is characterized by extensive genetic heterogeneity [1], and is divided into four groups, M (major), O (outlying), N (new or non-M, non-O) and P (pending). The viruses in group M are further classified into many subtypes and circulating recombinant forms (CRFs). Among them, subtypes A, B, C, D and G, as well as CRF01_AE and CRF02_AG, are the major subtypes and CRFs, which are responsible for the worldwide HIV-1 pandemic [2]. While subtype B of HIV-1 is the predominant subtype in the Americas, Europe and Australia, there is a growing epidemic of non-B subtypes and CRFs in Africa and Asia. CRF01_AE is prevalent throughout Southeast Asia [2], and is responsible for more than 80% of infection cases in Thailand [3].

The heterogeneity of the HIV-1 genome is mainly attributed to the error-prone nature of viral reverse transcriptase [4]. In the reverse transcription process, a G to A hypermutation is introduced into proviral DNA by the APOBEC3 family of cytosine deaminases [5], [6], [7], [8]. Although several lines of evidence have demonstrated that APOBEC3 family protein-mediated hypermutation plays an important role in the host defense mechanism against HIV-1 in a clinical setting [9], [10], [11], [12], [13], a sub-lethal level of APOBEC3 activity probably affect viral evolution, which facilitates viral escape from immune responses and antiretroviral therapy (ART) [14], [15].

The envelope glycoproteins (Env), gp120 and gp41, of HIV-1 play a central role in viral transmission to target cells, and mediate attachment and incorporation of the virus into the cells through specific interaction with the CD4 receptor and chemokine receptors. In addition, Env is a major target of humoral immune responses against HIV-1 [16], [17]. Env gp120 and gp41 are the most variable HIV-1 proteins with typical intersubtype and intrasubtype differences soaring to 35% and 20%, respectively [1]; therefore, the humoral immune responses against Env potentially somewhat vary among different subtypes and CRFs. For example, the replication of many clinical isolates of subtype B, C and D is neutralized by a human monoclonal antibody against the CD4 binding site (CD4BS) of Env gp120, IgG1 b12, which was established from an HIV-1 subtype B-infected individual [18], while most CRF01_AE viruses are resistant to IgG1 b12-mediated neutralization [19], [20], [21]. In addition, recently established, broadly neutralizing human monoclonal antibodies derived from HIV-1 subtype A-infected individuals recognize conserved regions of the V2 and V3 regions [22], while several broadly neutralizing, human or murine monoclonal antibodies elicited by the HIV-1 subtype B antigen recognize CD4BS or V3 region of Env gp120 [18], [23], [24], [25], [26], [27]. These results imply that the Env of different subtypes and CRFs show different antigenicity.

The introduction of mutations into HIV-1 Env, including those involved in the N-linked glycosylation of particular amino acid residues, leads to a reduction of the susceptibility to neutralizing antibodies [28], [29], [30]; therefore, the mutation-driven evolution of HIV-1 Env plays an important role in conferring viral escape from humoral immune responses. In order to study the evolutionary pattern of CRF01_AE Env under host immune pressure, we examined the changes of HIV-1 Env amino acid sequences derived from chronically CRF01_AE-infected Thai patients over 3 years.

## Methods

### Ethics statement

This study was conducted with the approval from the ethics committee of the Department of Medical Sciences, Ministry of Public Health of Thailand and with written informed consent from the patients.

### Study participants and sample collection

Peripheral blood samples were collected every 3 months from April 2008 to January 2011, namely 12 times in total, from 9 drug-naïve, HIV-1-infected patients as well as from 6 HIV-1-infected patients on ART. All patients were infected with HIV-1 CRF01_AE viruses [31], and were negative for hepatitis B and C viruses at the time of enrollment. Among 9 drug-naive patients, 4 patients started ART during the project.

### Measurement of CD4 count and viral load

As clinical markers, the CD4 count and viral load of the patients were monitored during the study period. The CD4 count was measured every 3 months by flow cytometric analysis at Chiangrai Prachanukoh Hospital, according to the manufacturer's protocol (Beckman Coulter, Fullerton, California, USA). In addition, the viral load was measured every 6 months as follows. Viral RNA was extracted from a plasma sample using High Pure System Viral Nucleic Acid (Roche, Basel, Switzerland). The viral load was then measured using the Cobas AmpliPrep/Cobas TaqMan HIV-1 version 5.1 Assay (Roche).

### Amplification of viral genomic fragment encoding a full length Env precursor gp160

Plasma was isolated from peripheral blood samples by centrifugation for 10 min at 2000 rpm. In addition, peripheral blood mononuclear cells (PBMC) were isolated by density gradient centrifugation using Ficoll-Paque (GE Healthcare, Buckinghamshire, UK). Prior to RNA extraction, viral particles were concentrated from 1-2 ml of plasma by ultracentrifugation for 1 hour at 65,000 rpm using TLA-100.3 rotor with Optima TLX ultracentrifuge (Beckman Coulter). RNA and DNA were then extracted from the concentrated viral particles and PBMC using the QIAamp viral RNA mini-kit and the QIAamp DNA blood mini-kit (Qiagen, Hilden, Germany), respectively. Viral RNA was reverse transcribed to cDNA using the SuperScript III First-Stand Synthesis kit (Invitrogen, Carlsbad, California, USA) with the reverse primer, K-env-R1, 5′-CCAATCAGGGAAGAAGCCTTG-3′ [corresponding to nucleotide (nt) 8736 to 8716 of CRF01_AE reference strain, CM240 (GenBank accession no. U54771)]. The HIV-1 genomic fragment, encoding full-length Env precursor gp160, Rev and Vpu as well as partial fragments of Tat and Nef, was then amplified by nested PCR using BIO-X-ACT DNA polymerase (Bioline, Luckenwalde, Germany) and one of two primer sets, as follows. As the first set of primers, N-env-F1; 5′-TTAGAGGAGCTTAAAAATGAAGC-3′ (nt 5193 to 5215) and N-env-R1; 5′-TTAAAAAGAAGCTAAGATCAAAAGC-3′ (nt 8638 to 8614) were used for the first PCR, and N-env-F2; 5′- GAATTGGGTGTCAACATAGCAGAATAGGC-3′ (nt 5344 to 5372) and N-env-R2; 5′- TATCTAGATCTTGAGATACTGCTCC-3′ (nt 8485 to 8461) were used for nested PCR. As the second set of primers, K-env-F1; 5′- CTAGAGCCCTGGAATCATCCG-3′ (nt 5419 to 5439) and K-env-R1; 5′- CCAATCAGGGAAGAAGCCTTG-3′ (nt 8736 to 8716) were used for the first PCR, and K-env-F2; 5′-CGAGGAACTCCTCAGAGCAG-3′ (nt 5563 to 5582) and K-env-R2; 5′-TCTTGTGCTCTCAGCCAGAC-3′ (nt 8549 to 8530) were used for nested PCR. If the PCR using the first set of primers failed to amplify viral genomic fragment, the second set of primers was used. The PCR conditions were as follows. For the 1st PCR using the first set of primers, one cycle of 1 min at 94°C for denaturation; 10 cycles of 20 sec at 94°C for denaturation, 30 sec at 48°C for annealing and 5 min at 68°C for extension; 20 cycles of 10 sec at 94°C for denaturation, 30 sec at 48°C for annealing and 5 min at 68°C for extension with cycle elongation of 10 sec for each cycle; and a final extension cycle of 10 min at 68°C were carried out. For the nested PCR using the first set of primers, one cycle of 1 min at 94°C for denaturation; 30 cycles of 10 sec at 94°C for denaturation, 30 sec at 52°C for annealing and 4 min at 68°C for extension with cycle elongation of 5 sec for each cycle; and a final extension cycle of 5 min at 68°C were carried out. For the 1st and nested PCR using the second set of primers, annealing temperatures were changed to 60°C and 58°C, respectively. If a viral gene fragment failed to be amplified from the cDNA generated from viral RNA even after multiple attempts, it was amplified instead from DNA extracted from PBMC. In order to examine the genomic fragment of the major viral population in a sample, PCR products amplified at the end-point dilution of cDNA or DNA templates were subjected to sequencing analysis.

### Sequencing and data analysis

Sequencing analysis of the amplified HIV-1 genomic fragment was carried out using the BigDye Terminator v3.1 Cycle Sequencing kit with an ABI PRISM 3130XL genetic analyzer (Applied Biosystems, Foster City, California, USA), and data were assembled using SeqScape v2.5 software (Applied Biosystems). The deduced amino acid sequences derived from an individual patient were then aligned with the viral sequence derived from the earliest sample collected in April 2008, using the ClustalW algorithm [32] with slight manual adjustment, followed by the numbering of amino acid residues according to their position in the HXB2 Env (Genbank accession no. K03455). Pairwise genetic distances between two amino acid sequences derived from an individual patient were calculated by using the p-distance model with 1,000 bootstrap replicates, conducted with the MEGA5 software package [33]. The diversity of each amino acid residue among 10 amino acid sequences derived from an individual was evaluated by calculating the Shannon index [34] with a program available at the website, http://www.gen-info.osaka-u.ac.jp/~uhmin/study/consensus/index.html. The nonsynonymous to synonymous substitution rate (dN/dS ratio) was estimated by calculating the numbers of synonymous substitutions per synonymous site (dS) and nonsynonymous substitutions per nonsynonymous site (dN) based on the Tamura-Nei method using the HyPhy program in the MEGA5 software package [33], [35]. Finally, the potential N-linked glycosylation (PNLG) site was evaluated using N-Glycosite (http://www.hiv.lanl.gov/).

### Nucleotide sequence accession numbers

The nucleotide sequences of the viral gene fragment encoding full-length Env precursor gp160 have been deposited in the GenBank database under accession numbers JN388081-JN388230.

## Results

### 

#### Sample collection from 15 Thai patients infected with HIV-1 CRF01_AE viruses

The molecular evolution of CRF01_AE Env, which was presumably driven by the selection pressure of humoral immune responses, was studied in this report. To this end, we periodically collected 10-12 viral sequences encoding full-length Env precursor gp160 for 3 years from 15 Thai patients chronically infected with HIV-1 CRF01_AE viruses, and performed computational analysis on 10 selected viral sequences derived from each patient (Table S1). We attempted to study viral sequences derived from plasma viral RNA; however, if a viral gene fragment failed to be amplified from viral RNA, that derived from proviral DNA was instead studied. Proviral DNA is generally considered to be more heterogeneous than viral RNA; however, the concordance of viral sequences between viral RNA and the major population of proviral DNA was observed previously [36], [37], [38]. Indeed, homogeneous viral sequences were amplified at the end-point dilution of proviral DNA samples (data not shown). The CD4 count and viral load were measured as clinical markers during the study (Tables S2 and S3). The CD4 counts of most patients were relatively stable, but fluctuated over 3 years (Table S2). In addition, the CD4 counts of 4 drug-naive patients gradually declined to <200 cells/mm^3^, and subsequently increased in response to ART (Table S2). The viral loads of the patients on ART were close to or under the detection limit throughout the study, whereas these of drug-naive patients gradually increased during the study (Table S3). These results suggested that the clinical conditions of most patients were relatively stable over 3 years, but those of some patients gradually deteriorated during the study.

### Variability of CRF01_AE Env gp120 and gp41 over 3 years

**Figure 1:**
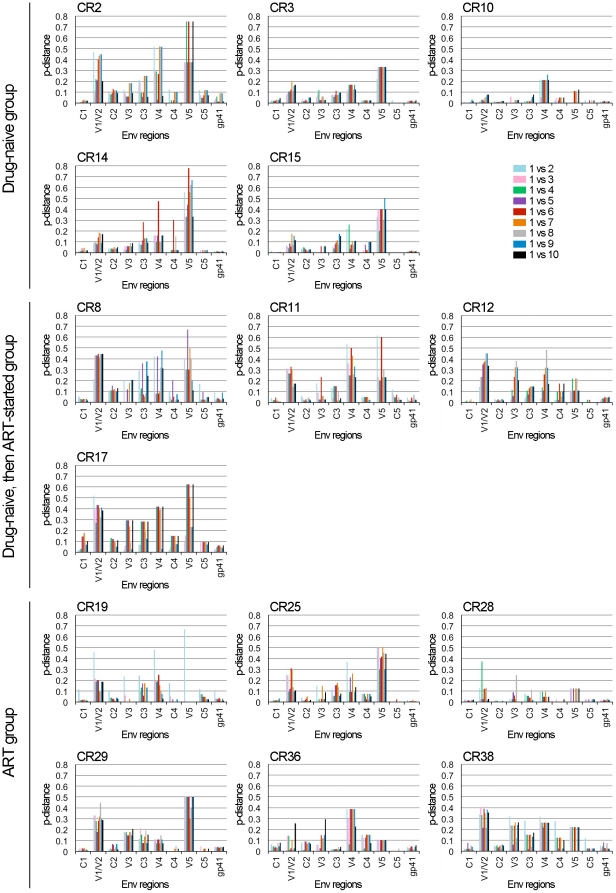
Variability of amino acid sequences in Env gp120 regions and gp41. Nine amino acid sequences derived from samples collected between July 2008 and January 2011 from an individual were aligned with the amino acid sequence derived from the earliest sample collected in April 2008 from a corresponding individual. Aligned sequences were then divided into variable and conserved regions of gp120 and gp41. Pairwise genetic distances (p-distance) between the sequences derived from the earliest and one of the later samples was determined as described in Methods. The data obtained for all Env regions were plotted together in chronological order of sampling, as indicated from 1 vs 2 to 1vs 10. Patient IDs, Env regions and the status of treatment are denoted above, below and beside the panels, respectively.

After the establishment of HIV-1 infection, the amino acid diversity of HIV-1 Env increases during the course of disease progression [39]. In order to evaluate the molecular evolution of the functional regions of CRF01_AE Env under the pressure of humoral immune responses, we studied the variability of amino acid sequences in the conserved and variable regions of Env gp120 as well as in gp41. To this end, pairwise genetic distances (p-distances) between the amino acid sequences of an Env region in the earliest and one of the later samples derived from an individual were determined, and were plotted together to compare the variability of each Env region over 3 years. The results showed that the pairwise genetic distances of the amino acid sequences in gp120 variable regions, V1-V5, were higher than in other regions, including the conserved regions, C1-C5, of gp120 and gp41, as expected (Fig. 1). The V5 region showed the highest variability in samples derived from 8 individuals, CR2, CR3, CR14, CR15, CR11, CR17, CR25 and CR29, whereas the V1/V2 or V4 region(s) showed higher variability than the V5 region in samples derived from 7 individuals, CR10, CR8, CR12, CR19, CR28, CR36 and CR38 (Fig. 1). V3 region showed relatively low variability among variable regions (Fig. 1). In addition, C3 showed the highest variability among the conserved regions of gp120 (Fig. 1). Pairwise genetic distances of amino acid sequences in 5 conserved regions are also shown in Figure S1. Finally, gp41 showed lower variability than gp120 in all samples (Fig. 1). There were two patterns of change in the amino acid variability of Env gp120 and gp41 over 3 years. One was a gradually increasing pattern (example; CR3, V1/V2 regions), while the other was a fluctuating pattern of variability during 3 years (example, CR2, C3 region) (Figs. 1 and S1).

### High amino acid diversity in the N-terminal part of C3 region and in entire V5 region

**Figure 2:**
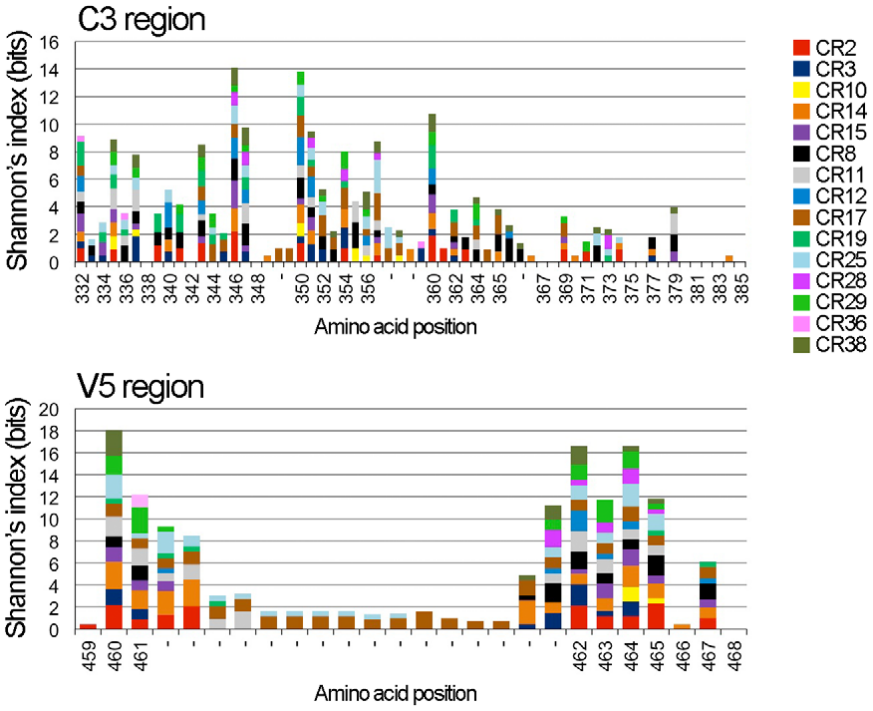
Amino acid residues with high diversity in the C3 and V5 regions of gp120. The Shannon index was calculated for each amino acid residue in the C3 and V5 regions of gp120 among 10 amino acid sequences derived from an individual, as described in Methods. The data obtained from 15 patient samples were then plotted together. Amino acid numbering is based on the HXB2 Env gp120.

C3 and V5 regions showed the highest variability among the conserved and variable regions of gp120, respectively (Figs. 1 and S1). We next attempted to determine amino acid residues with high diversity within these regions by calculating the Shannon index of diversity. The Shannon index is a diversity index used to measure diversity in categorical data. We calculated the index for each amino acid residue in the C3 and V5 regions of gp120 among 10 viral sequences derived from an individual. The results showed that the N-terminal, but not C-terminal part of the C3 region contained amino acid residues with Shannon index values of 0.5-2.5 in many samples, indicating that high amino acid diversity was observed in the N-terminal part of the C3 region (Fig. 2). In addition, most amino acid residues, except a few amino acid residues at N- and C-termini, of the V5 region showed high diversity (Fig. 2). A large amino acid insertion was found in the middle part of the V5 region of gp120 in a few samples, and these amino acid residues also showed high diversity (Fig. 2).

### Variations in the length of gp120 variable regions and the number of PNLG sites

**Figure 3:**
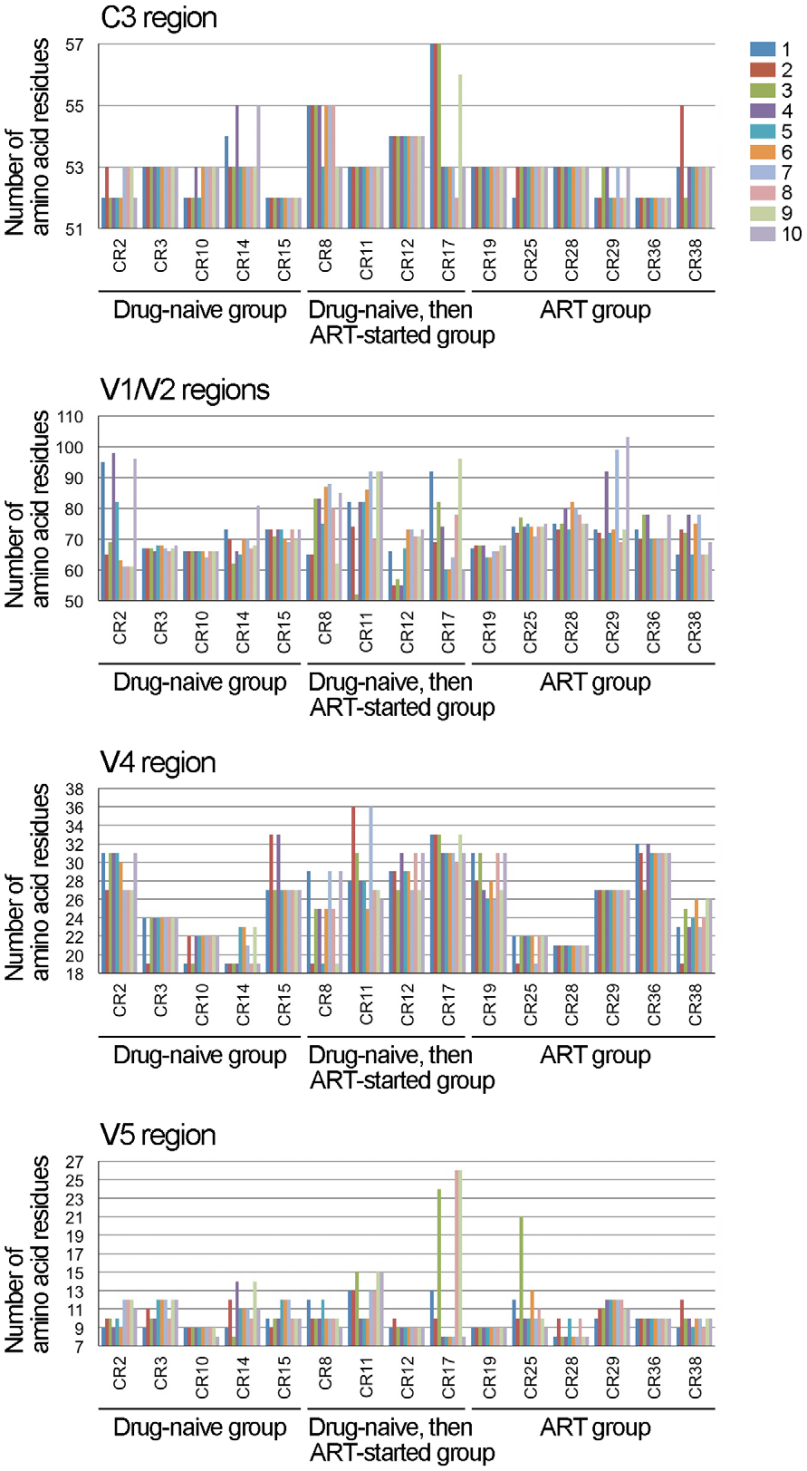
Changes in the number of amino acid residues in the gp120 regions. The numbers of amino acid residues in the gp120 regions were counted manually for 10 amino acid sequences derived from an individual. The data obtained from 15 patient samples were then plotted together in chronological order of sampling, as indicated from 1 to 10. Env regions are denoted above the panels, whereas patient IDs and the status of treatment are denoted below the panels.

**Figure 4:**
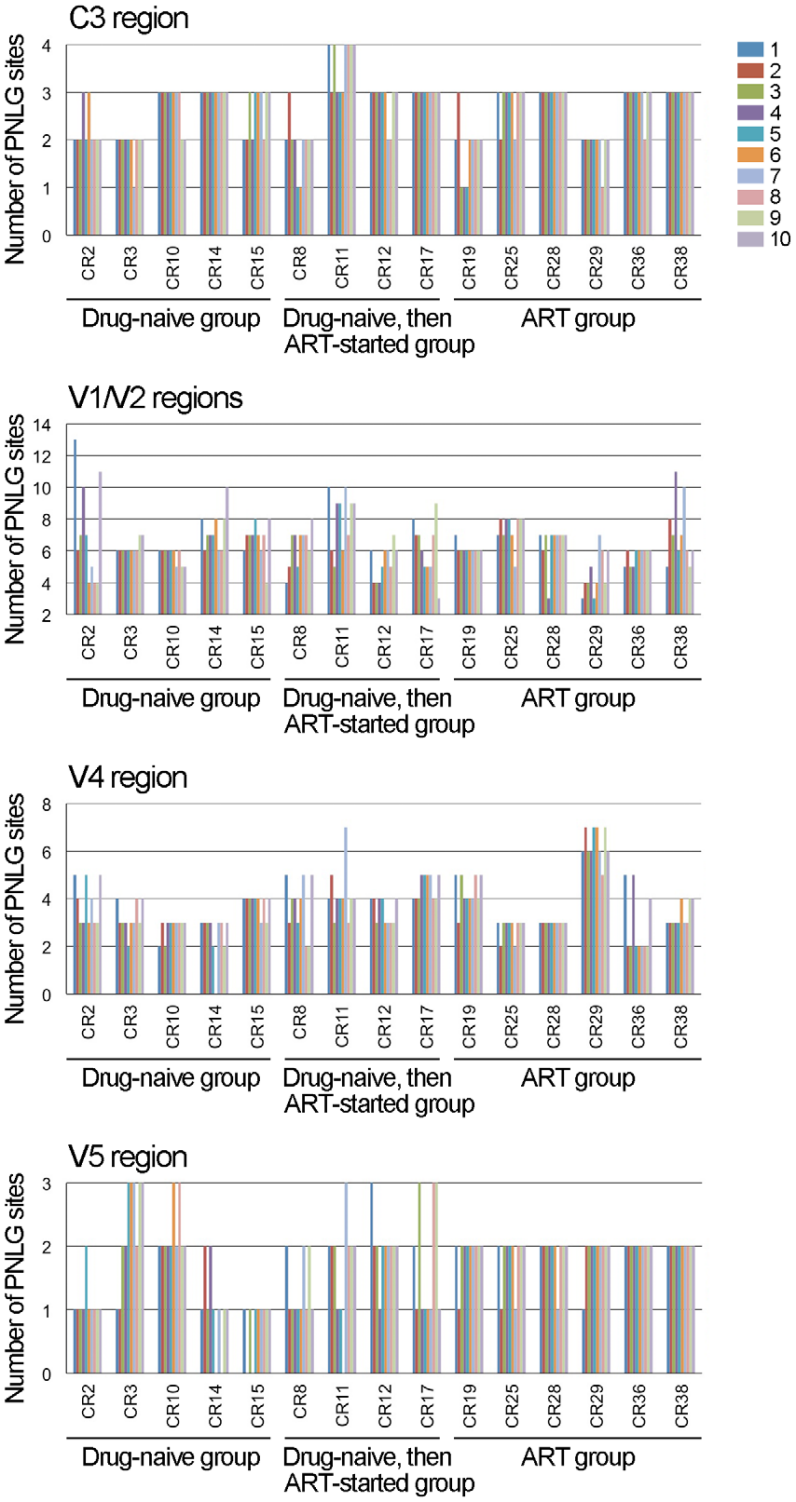
Changes in the number of PNLG sites in the gp120 regions. PNLG sites in the gp120 regions were evaluated using N-Glycosite, and the number of the sites was then counted manually for 10 amino acid sequences derived from an individual. The data obtained from15 patient samples were then plotted together in chronological order of sampling, as indicated from 1 to 10. Env regions are denoted above the panels, whereas patient IDs and the status of treatment are denoted below the panels.

The length of the variable regions of gp120 as well as the N-linked glycosylation of particular amino acid residues affect the protein structure and lead to changes in the neutralization susceptibility of HIV-1 Env [17], [30], [40]; therefore, these factors are important for the molecular evolution of HIV-1 Env. We studied the changes in the numbers of amino acid residues and PNLG sites in the functional regions of gp120 and gp41. Average numbers of amino acid residues and PNLG sites in each Env region are shown in Table 1. The amino acid numbers did not significantly change over 3 years in the C1, C2, V3, C4 and C5 regions of gp120 as well as in gp41 (data not shown). In contrast, the numbers of amino acid residues in the V1/V2, C3, V4 and V5 regions of gp120 varied among samples derived from different individuals, and the numbers changed over 3 years (Fig. 3). A moderate correlation was observed between the variability and the chronological change of amino acid numbers in V1/V2, V4 and V5 regions (Figs. 1 and 3). Namely, if the chronological changes, either increasing or decreasing, in amino acid numbers were frequent (Fig. 3), the variability of the Env regions were high in some samples (examples; V1/V2 regions of CR2, V4 region of CR11, V5 region of CR14) (Fig. 1). The number of PNLG sites changed significantly in the V1/V2 and V4 regions of gp120 over 3 years, whereas only a few chronological changes were observed in the number of PNLG sites in the C1, C2, V3, C3, C4 and V5 regions of gp120 as well as in gp41 (Fig. 4 and data not shown). A moderate correlation was again observed between the variability and the chronological change in the number of PNLG sites in the V1/V2, C3 and V4 regions of gp120 (Figs. 1, S1 and 4).

**Table 1:** Average numbers of amino acid residues and potential N-linked glycosylation (PNLG) sites in Env regions.

Env regions	Number of amino acid residues*	Number of PNLG sites*
gp120		
C1	129.9±0.7	1.9±0.5
V1/V2	72.4±9.1	6.4±1.6
C2	99.9±1.1	6.4±0.8
V3	34.1±0.3	0.7±0.4
C3	53.1±1.0	2.6±0.6
V4	26.0±4.3	3.6±1.2
C4	40.0±0	1.3±0.5
V5	10.6±2.8	1.7±0.7
C5	42.0±0	0
gp41	352.8±2.6	4.2±0.7

*The number of amino acid residues in an Env region was counted manually. PNLG sites were evaluated using N-Glycosite, and then counted manually. The means and standard deviations of the data calculated using 150 Env sequences derived from 15 individuals are shown.

### Detection of the amino acid residues potentially under positive selection

**Figure 5:**
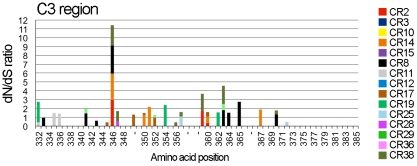
Amino acid residues potentially under positive selection in the C3 region of Env gp120. The dN/dS ratio was estimated for each amino acid residue in the C3 region of gp120 among 10 amino acid sequences derived from an individual, as described in Methods. The data obtained from 15 patient samples were then plotted together. Amino acid numbering is based on the HXB2 Env gp120.

We next attempted to determine the amino acid residues in gp120 and gp41 under positive selection. To this end, the dN/dS ratio was calculated, and an amino acid residue with a ratio greater than 1 was considered to be potentially under positive selection. Since insertion and deletion mutations were frequently introduced into the V1/V2, V4 and V5 regions of gp120 over 3 years in many samples (Fig. 3), we failed to estimate reliable dN and dS values following the alignment of amino acid sequences for these variable regions. In contrast, the dN/dS ratios of the amino acid residues in the V3 and five conserved regions of gp120 as well as in gp41 were successfully determined. We found that the dN/dS ratios were greater than 1 at 6, 5, 3, 17, 7, 0 and 9 amino acid residues in the C1, C2, V3, C3, C4 and C5 regions of gp120 and gp41, respectively, in samples derived from at least one individual (Table 2). The C3 region of gp120 contained several amino acid residues with a dN/dS ratio greater than 1 (Table 2). The dN/dS ratios of amino acid residues in C3 region are shown in Figure 5. Our results showed that several amino acid residues in the N-terminal part of C3 region showed high diversity (Fig. 2), and some of these amino acid residues were potentially under positive selection (Fig. 5).

**Table 2:** Amino acid residues potentially under positive selection.*

Env regions	Amino acid residue (dN/dS ratio, patient ID)**
gp120	
C1	28 (1.03, CR28), 31 (2.43, CR19), 60 (1.05, CR36), 84 (6.24, CR17),85 (1.14, CR14), 119 (1.46, CR17)
C2	212 (1.28, CR2), 240 (2.33, CR38), 261 (1.01, CR19), 263 (2.31, CR8),290 (1.28, CR19)
V3	310 (1.54, CR8), 315 (1.07, CR28), 320 (2.57, CR36)
C3	332 (2.32, CR19), 335 (1.47, CR11), 336 (1.42, CR11), 341 (1.44, CR8),346 (3.00, CR2; 3.20, CR8; 2.96, CR14; 2.30, CR38), 347 (1.23, CR38),349′ (1.28, CR17),*** 350 (1.21, CR14), 351 (2.16, CR14), 354 (2.37, CR19),359 (1.75, CR2; 1.92, CR38), 362 (1.54, CR19), 363 (1.79, CR8; 2.08, CR38),364 (1.50, CR8), 365 (2.75, CR8), 367 (1.89, CR14), 370 (1.32, CR8)
C4	421 (1.53, CR14), 439 (1.42, CR38), 440 (16.34, CR12), 442 (1.99, CR14; 1.50, CR29; 1.50, CR38), 447 (1.21, CR14), 450 (1.10, CR14), 454 (1.07, CR19)
C5	ND****
gp41	535 (1.61, CR38), 585 (1.78, CR36), 600 (1.03, CR8), 647 (1.01, CR17),658 (1.76, CR2), 718 (1.60, CR17), 776 (1.93, CR2), 804 (3.14, CR19),853 (1.35, CR17; 1.31, CR28)

*dN/dS ratio was determined as described in Methods, and the amino acid residue at which dN/dS ratio was greater than 1 was considered to be potentially under positive selection.

**Amino acid numbering is according to that of HXB2 Env. dN/dS ratio and Patient ID are shown in parentheses.

***Inserted amino acid residue between position 349 and 350.

****Not detected.

### Involvement of APOBEC3 activity in amino acid substitutions due to the positive selection of Env

The sub-lethal level of APOBEC3 activity is proposed to be involved in viral evolution; therefore, we attempted to study the possible involvement of APOBEC family protein-mediated G to A mutation in the positive selection of Env amino acid residues. APOBEC3G and APOBEC3F are involved in GG-AG [41] and GA-AA mutations [42], respectively. We manually detected GG-AG, GA-AA, GC-AC and GT-AT mutations, and found one of these mutation patterns 44 times over 3 years at 21 out of 47 amino acid residues with a dN/dS ratio greater than 1 (45%) in the C1, C2, V3, C3, C4 and C5 regions of gp120 and gp41 (Table 3). G to A mutations were more frequently detected in samples derived from individuals on ART, including CR17 and CR19, compared to samples derived from drug-naive individuals including CR2 and CR14 (Table 3), suggesting the accumulation of viruses with G to A mutations under therapeutic pressure and limited viral replication. Nevertheless, these results suggested that APOBEC3 activity was involved, at least in part, in mutations due to the positive selection of Env gp120 and gp41.

**Table 3:** Association of positive selection and potential APOBEC3-related mutation.

Env regions	Amino acid residue*	Patient ID	G to A mutation**
gp120			
C1	28	CR25	GG to AG x3***
	31	CR19	GA to AA
	60	CR36	GG to AG
	84	CR17	GA to AA
	85	CR8	GG to AG
C2	290	CR19	GA to AA x2, GT to AT
		CR29	GA to AA x2, GT to AT
C3	332	CR11	GA to AA x2
		CR19	GT to AT
	335	CR11	GA to AA x2, GG to AG
	346	CR2	GA to AA
	347	CR38	GA to AA x3
	350	CR14	GG to AG, GA to AA
		CR17	GG to AG x2
	362	CR19	GA to AA
C4	439	CR19	GT to AT
	440	CR12	GG to AG
		CR17	GG to AG
	442	CR29	GA to AA
		CR38	GA to AA
	454	CR19	GA to AA
gp41	535	CR38	GA to AA x2
	585	CR36	GT to AT x2
	600	CR8	GA to AA x3
	647	CR17	GG to AG
	658	CR2	GA to AA x2

*Amino acid residues are numbered according to HXB2 Env protein.

**Mutations, GG to AG, GA to AA, GC to AC and GT to AT, at amino acid residues with dN/dS ratio >1 were manually detected.

***GG to AG mutation was sequentially detected 3 times among 10 Env amino acid sequences derived from an individual.

## Discussion

The molecular evolution of CRF01_AE Env was studied using viral gene fragments periodically collected from 15 chronically HIV-1-infected Thai patients over 3 years. It was previously reported for subtype B Env that the V1/V2 regions of gp120 are under positive selection *in vivo* [43], and the expansion of V1/V2 regions along with the accumulation of PNLG sites reduces the susceptibility of viruses to autologous neutralizing antibody [28]. The V3 region of subtype B gp120 shows strong immunogenicity, and several neutralizing monoclonal antibodies have been established [23], [24], [27]. In addition, the positive selection of amino acid residues in the V3 region is reported [44], [45], [46]. In contrast, the V3 region of subtype C gp120 is conserved, and the molecular evolution of C3-V4 regions is observed under the selection pressure of autologous serum antibodies [29]. In addition, the V5 region, cooperating in part with the V3 region, of subtype C gp120 is involved in generating an escape variant against humoral immune responses [47]. We first attempted to identify the region of CRF01_AE Env that showed high variability over 3 years, and found that the V1/V2, V4 and V5 regions of CRF01_AE gp120 showed high variability (Fig. 1). The gp120 region that showed highest variability was different among patients, suggesting that the V1/V2, V4 or V5 regions differentially counteracts with humoral immune responses. In contrast to these regions, the V3 region showed relatively low variability among the variable regions of gp120 (Fig. 1). The vast majority of CRF01_AE Env clones derived from plasma samples were CCR5-tropic [48]. In addition, CCR5-tropic CRF01_AE viruses with a low positive charge in the V3 region show low neutralization susceptibility to anti-V3 antibodies [49]. Considering these reports together, the V3 region of CCR5-tropic CRF01_AE viruses may not be under strong selection pressure of humoral immune responses in chronically infected, asymptomatic patients.

Molecular evolution of the C3 region of subtype C gp120 was previously reported [50]. An alpha helix domain located in the N-terminal part of the C3 region of subtype C gp120 shows high variation and is under positive selection, whereas that of subtype B gp120 is relatively conserved [1], [51]. In addition, the quaternary structure of this region differs between subtype B and C Env [52], and the C3 region of subtype C, but not of subtype B gp120 is suggested to be under immune pressure. Furthermore, mutations in the C3 region, cooperating with those in the variable regions, of subtype C gp120 affect the neutralization susceptibility of viruses to autologous neutralizing antibodies [53]. Our results showed that the C3 region of CRF01_AE gp120, similar to that of subtype C gp120, showed high amino acid variation (Figs. 1 and S1), and was suggested to be under the selection pressure of autologous immune responses.

Viral evolution in patients on ART has been reported previously [45]. Consistent with the previous report, high variability was observed in the variable regions of gp120 derived not only from drug-naïve patients, but also from patients on ART (Fig. 1). We observed not only a gradually increasing pattern, but also a fluctuating pattern in the chronological change in the variability of Env amino acid sequences over 3 years (Fig. 1), indicating two possibilities: that the variability was generated by the evolution of a trace of escape mutants against ART or was due to viral quasispecies which acquired divergence prior to the onset of ART. The frequent appearance of stop codons in the C2, V3 and C3 regions of gp120 in proviral DNA derived from successfully treated patients is reported [54]. In contrast to the previous report, premature stop codons were not frequently detected in Env gp120 and gp41 derived from patients on ART (data not shown). A possible explanation for this discrepancy might be the difference in efficacy, including the potency and the level of adherence, of ART between patients enrolled in our and other studies.

Potential APOBEC3 family protein-mediated G to A mutations were detected among the mutations due to the positive selection of several Env amino acid residues (Table 3), suggesting the role of a sub-lethal level of APOBEC3 activity in viral evolution. APOBEC3G introduces missense or nonsense mutations into viral DNA during the RT process and leads to a diminishment of viral infectivity, whereas HIV-1 Vif counteracts this activity [5], [7], [8]; therefore, we consider that an approach to inhibit Vif function or stimulate APOBEC3 activity has potential for therapy.

Finally, HIV-1 Env, gp120 and gp41 are the major targets among viral proteins of humoral immune responses to viral infection; therefore, they are candidates of vaccine antigens. In order to develop an effective vaccine, it is important to understand the immunological characteristics of target viral proteins; however, information regarding the molecular evolution of CRF01_AE Env is still limited. CRF01_AE is a major circulating recombinant form of HIV-1 prevalent in Southeast Asia, including Thailand, and HIV vaccine trials have been conducted in Thailand [55], [56]. We hope that our results may provide useful information for understanding the immunological characteristics of CRF01_AE Env as well as for designing effective vaccine antigens.

## Supporting Information

Figure S1 Variability of amino acid sequences in the conserved regions of Env gp120. Pairwise genetic distances (p-distance) was determined, as described in the legend to Figure 1. Patient IDs, Env regions and the status of treatment are denoted above, below and beside the panels, respectively.
(TIF)

Table S1 Sampling information.
(DOC)

Table S2 Changes in the CD4 count of study participants.
(DOC)

Table S3 Changes in the viral load of study participants.
(DOC)
