# Supplementary figures and images for: Molecular Evolution of HIV-1 CRF01_AE Env in Thai Patients
Source: PLoS One. 2011 Nov 2;6(11):e27098. doi: 10.1371/journal.pone.0027098 (PMC3206936; doi:10.1371/journal.pone.0027098)

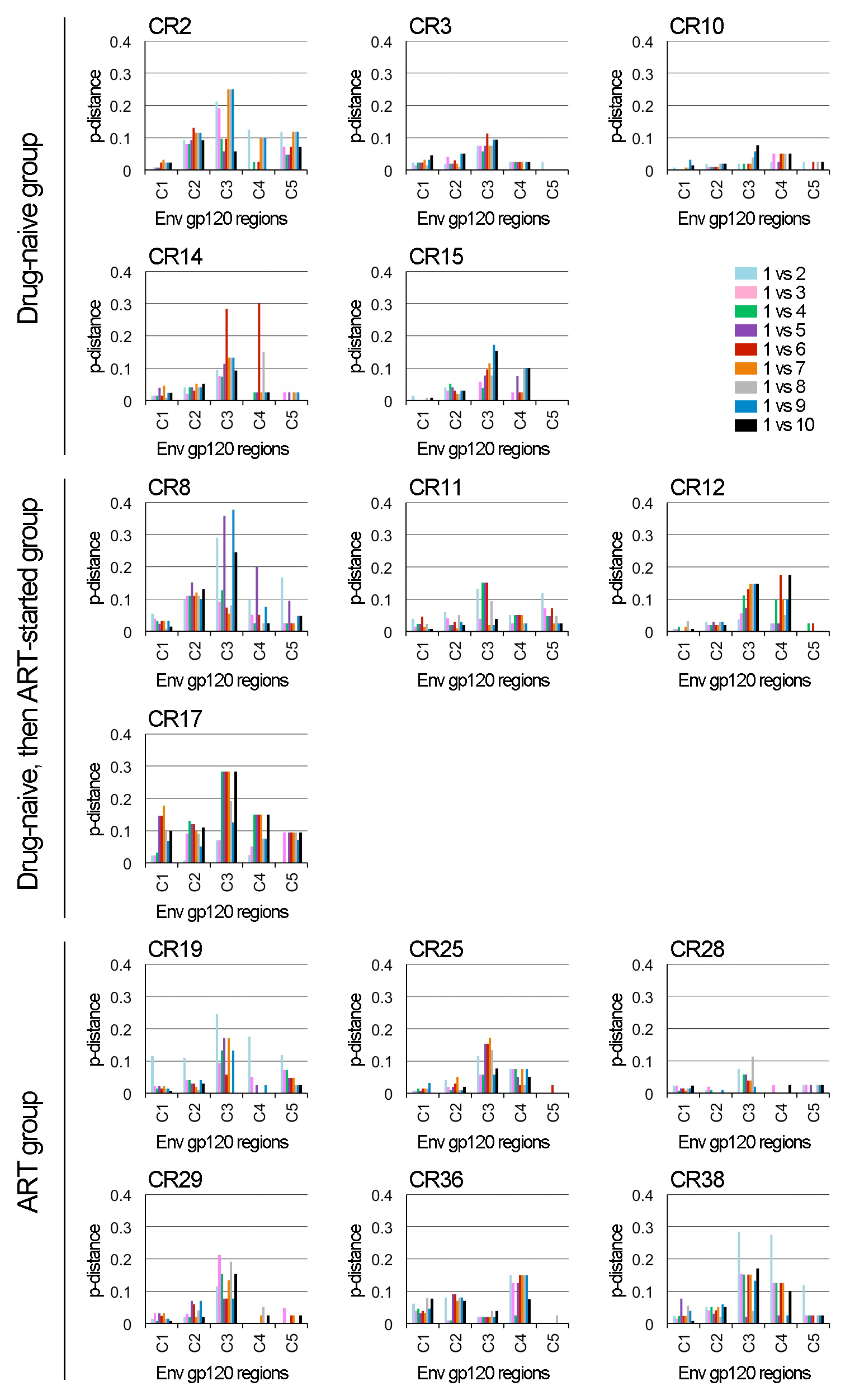

Supplement: Figure S1 — Variability of amino acid sequences in the conserved regions of Env gp120. Pairwise genetic distances (p-distance) was determined, as described in the legend to Figure 1. Patient IDs, Env regions and the status of treatment are denoted above, below and beside the panels, respectively. (TIF) [file pone.0027098.s001.tif]
