# Supplementary material for: Molecular Evolution of HIV-1 CRF01_AE Env in Thai Patients
Source: PLoS One. 2011 Nov 2;6(11):e27098. doi: 10.1371/journal.pone.0027098 (PMC3206936; doi:10.1371/journal.pone.0027098)
Supplement: Table S1 — Sampling information. (DOC) [file pone.0027098.s002.doc]

**Supplementary Table 1.** Sampling information.

|  | 2008 | | | 2009 | | | | 2010 | | | | 2011 |
| --- | --- | --- | --- | --- | --- | --- | --- | --- | --- | --- | --- | --- |
| Patient ID | April | July | October | January | April | July | October | January | April | July | October | January |
| Drug-naive group | | | | | | | | | | | | |
| CR2 | 1D * | 2R | - | 3R | 4R | - | 5R | 6R | 7R | 8R | 9R | 10R |
| CR3 | 1R | 2R | - | - | 3D | 4R | 5R | 6R | 7R | 8R | 9R | 10R |
| CR10 | 1D | x | 2D | 3D | 4R | 5R | 6R | 7D | 8R | x | 9D | 10R |
| CR14 | 1R | 2D | x | 3D | x | 4D | 5R | 6D | 7R | 8R | 9D | 10D |
| CR15 | 1R | 2R | - | - | 3R | 4R | 5R | 6R | 7R | 8R | 9R | 10R |
| Drug-naive, then ART-started group | | | | | | | | | | | | |
| CR8 | 1R | 2R | - | x | 3R | 4R | 5R | 6D +1 | 7D +1 | 8D +1 | 9D +1 | 10D +2 |
| CR11 | 1R | 2R | - | 3D | 4R | 5R | 6R | 7R | 8D +1 | - | 9D +1 | 10D +1 |
| CR12 | 1R | 2R | - | - | 3R | 4R | 5R | 6D | 7R +1 | 8D +3 | 9D +3 | 10D +3 |
| CR17 | 1R | 2R | - | - | 3D +1 | 4D +1 | 5D +1 | 6D +1 | 7D +1 | 8D +1 | 9D +1 | 10D +1 |
| ART group | | | | | | | | | | | | |
| CR19 | 1D +1 | 2D +1 | - | - | 3D +1 | 4D +1 | 5D +1 | 6D +1 | 7D +1 | 8D +1 | 9D +1 | 10D +2 |
| CR25 | 1D +2 | 2D +2 | - | - | 3D +2 | 4D +2 | 5D +2 | 6D +2 | 7D +2 | 8D +2 | 9D +2 | 10D +2 |
| CR28 | 1D +4 | 2D +4 | - | - | 3D +4 | 4D +4 | 5D +4 | 6D +4 | 7D +4 | 8D +4 | 9D +4 | 10D +4 |
| CR29 | 1D +1 | 2D +1 | - | - | 3D +1 | 4D +1 | 5D +1 | 6D +1 | 7D +1 | 8D +1 | 9D +1 | 10D +2 |
| CR36 | 1D +1 | 2D +1 | - | - | 3D +1 | 4D +1 | 5D +1 | 6D +1 | 7D +1 | 8D +1 | 9D +1 | 10D +2 |
| CR38 | 1D +1 | 2D +1 | - | - | 3D +1 | 4D +1 | 5D +1 | 6D +1 | 7D +1 | 8D +1 | 9D +1 | 10D +2 |

* Viral RNA-derived cDNA (R) or proviral DNA (D), which were collected between April 2008 and January 2011, were subjected to PCR amplification and sequencing analysis of HIV-1 *env* gene. Ten selected sequencing data were subjected to computational analysis, as indicated. (-) denotes sample not selected for analysis, while (x) denotes that the sample failed in PCR amplification and/or sequencing. Samples collected from patients on ART are denoted as (+). The regimen was as follows: GPOvir (d4T/3TC/NVP) (+1), GPOZ/GPOvir2 (AZT/3TC/NVP) (+2), AZT/ddI/EFV (+3) or d4T/3TC/EFV (+4).
