# Supplementary material for: Molecular Evolution of HIV-1 CRF01_AE Env in Thai Patients
Source: PLoS One. 2011 Nov 2;6(11):e27098. doi: 10.1371/journal.pone.0027098 (PMC3206936; doi:10.1371/journal.pone.0027098)
Supplement: Table S2 — Changes in the CD4 count of study participants. (DOC) [file pone.0027098.s003.doc]

**Supplementary Table 2.** Changes in the CD4 count of study participants.

|  | 2008 | | | 2009 | | | | 2010 | | | | 2011 |
| --- | --- | --- | --- | --- | --- | --- | --- | --- | --- | --- | --- | --- |
| Patient ID | April | July | October | January | April | July | October | January | April | July | October | January |
| Drug-naive group | | | | | | | | | | | | |
| CR2 | 1,116* | 1,129 | 1,113 | 1,057 | 1,109 | 805 | 1,174 | 981 | 4,259 | 3,747 | 3,603 | 1,902 |
| CR3 | 685 | 725 | 680 | 595 | 470 | 635 | 583 | 492 | 555 | 546 | 510 | 376 |
| CR10 | 372 | 385 | 550 | 416 | 373 | 239 | 297 | 400 | 403 | 287 | 302 | 261 |
| CR14 | 310 | 582 | 487 | 506 | 511 | 655 | 569 | 360 | 564 | 439 | 360 | 341 |
| CR15 | 281 | 472 | 538 | 455 | 565 | 342 | 450 | 337 | 582 | 382 | 386 | 365 |
| Drug-naive, then ART-started group | | | | | | | | | | | | |
| CR8 | 506 | 603 | 440 | 391 | 328 | 262 | 152 | 386 | 102 | 289 | 319 | 337 |
| CR11 | 328 | 321 | 546 | 359 | 496 | 320 | 285 | 173 | 267 | 338 | 362 | 209 |
| CR12 | 321 | 351 | 306 | 727 | 398 | 304 | 350 | 272 | 657 | 301 | 419 | 397 |
| CR17 | 239 | 160 | 316 | 507 | 477 | 465 | 220 | 421 | 405 | 433 | 352 | 370 |
| ART group | | | | | | | | | | | | |
| CR19 | 544 | 388 | 611 | 886 | 606 | 697 | 581 | 627 | 1011 | 747 | 786 | 761 |
| CR25 | 403 | 404 | 380 | 432 | 613 | 433 | 369 | 286 | 369 | 462 | 457 | 498 |
| CR28 | 379 | 591 | 560 | 411 | 522 | 321 | 605 | 347 | 426 | 379 | 500 | 521 |
| CR29 | 364 | 300 | 450 | 585 | 327 | 464 | 391 | 303 | 421 | 500 | 431 | 362 |
| CR36 | 259 | 279 | 395 | 293 | 437 | 443 | 346 | 262 | 263 | 335 | 320 | 311 |
| CR38 | 251 | 243 | 324 | 332 | 321 | 337 | 307 | 296 | 301 | 369 | 193 | 193 |

*The CD4 count was measured by flow cytometric analysis. The number of cells per mm^3^ (cells/ mm^3^) is shown.
