# Supplementary material for: Molecular Evolution of HIV-1 CRF01_AE Env in Thai Patients
Source: PLoS One. 2011 Nov 2;6(11):e27098. doi: 10.1371/journal.pone.0027098 (PMC3206936; doi:10.1371/journal.pone.0027098)
Supplement: Table S3 — Changes in the viral load of study participants. (DOC) [file pone.0027098.s004.doc]

|  | 2008 | | 2009 | | 2010 |
| --- | --- | --- | --- | --- | --- |
| Patient ID | October | April | October | April | October |
| Drug-naive group | | | | | |
| CR2 | 2,410* | 10,700 | 2,160 | 48,700 | 204,000 |
| CR3 | 2,410 | 3,630 | 2,340 | 7,510 | 9,600 |
| CR10 | 228 | 41 | 144 | 70 | 11,600 |
| CR14 | 2,600 | 2,520 | 12,100 | 8,670 | 8,650 |
| CR15 | 682 | 838 | 1,030 | 3,150 | 386 |
| Drug-naive, then ART-started group | | | | | |
| CR8 | 273,000 | 255,000 | 1,220,000 | 80 | 79 |
| CR11 | 336,000 | 78,800 | 80,900 | 91 | <40 |
| CR12 | 19,800 | 125,000 | 90,700 | 1,110 | <40 |
| CR17 | 130 | <40 | <40 | <40 | <40 |
| ART group | | | | | |
| CR19 | <47 | <40 | <40 | <40 | <40 |
| CR25 | <47 | <40 | <40 | <40 | <40 |
| CR28 | <47 | <40 | <40 | <40 | <40 |
| CR29 | <47 | <40 | <40 | <40 | <40 |
| CR36 | <47 | <40 | <40 | <40 | <40 |
| CR38 | <47 | <40 | <40 | <40 | <40 |

**Supplementary Table 3.** Changes in the viral load of study participants.

*Viral RNA was extracted from a plasma sample, and the viral load was then measured using the Cobas AmpliPrep/Cobas TaqMan HIV-1 version 5.1 Assay (Roche). The copy number of viral RNA per ml (RNA copy/ ml) is shown.
